# Advances in Magnetic Nanocomposite Adsorbents for Water Remediation: Design, Performance, and Challenges

**DOI:** 10.3390/nano15181425

**Published:** 2025-09-16

**Authors:** Mingyu Yan, Chao Sun, Keying Sun, Derui Chen, Longbin Xu, Shunyu Han, Xinyu Li

**Affiliations:** 1 College of Engineering, Materials and Chemical Engineering, Yanbian University, Yanji 133002, China; 2024050098@ybu.edu.cn (M.Y.); 2024050102@ybu.edu.cn (K.S.); 2023050082@ybu.edu.cn (D.C.); 2 Department of Physics, Jilin University, Changchun 130012, China; sunc344@jlu.edu.cn; 3 Department of Chemistry, College of Science, Yanbian University, Yanji 133002, China; 4 Department of Polymer Materials & Engineering, College of Engineering, Yanbian University, Yanji 133002, China

**Keywords:** adsorption, nanomaterials, magnetic, composites, recovery, wastewater

## Abstract

Water pollution by heavy metals, dyes, and antibiotics is a serious environmental problem. Efficient and recyclable adsorbents are needed. Magnetic nanocomposite adsorbents (MNAs) offer a promising solution. They combine magnetic nanoparticles with various carriers. This gives them high adsorption capacity and easy magnetic separation. This review covers recent progress in MNAs. We focus on three carrier types: carbon-based materials, inorganic minerals, and natural polymers. We analyze common synthesis methods like co-precipitation and hydrothermal synthesis. The synergy between components enhances pollutant removal, however, challenges remain. These include poor selectivity in mixed pollutants and difficult large-scale production. Stability during reuse is also a concern. Future work should aim for greener synthesis and better stability. This review provides useful insights for designing high-performance MNAs for water treatment.

## 1. Introduction

The rapid development of industrialization and urbanization has led to increasingly severe water pollution issues. Pollutants in water bodies, notably heavy metal ions, organic dyes, and pharmaceutical antibiotics, pose significant threats to ecosystems and human health [1,2]. Although traditional water treatment technologies exhibit certain efficacy, they face challenges such as elevated costs and substantial risks of secondary pollution [3]. Adsorption technology is widely employed for the removal of aqueous pollutants due to its simplicity, high efficiency, operational convenience, and suitability for large-scale industrial applications [4]. However, conventional adsorbent materials often suffer from limitations such as restricted adsorption capacity and, more critically, difficulties in efficient separation and recovery from water. Typically, energy-intensive centrifugation or filtration steps are required for their retrieval, which complicates the process and may even lead to secondary pollution [5]. In this context, magnetic adsorbents have emerged as a prominent research focus in the field of water remediation. Their key advantage lies in their unique magnetic responsiveness, which enables rapid solid-liquid separation simply by applying an external magnetic field. This facilitates facile magnetic recovery, effectively preventing secondary pollution and significantly enhancing the operational efficiency of the treatment process.

Magnetic adsorbents are materials that exhibit magnetic responsiveness. Based on design principles, magnetic adsorbents can be classified into intrinsic magnetic materials and composite magnetic materials [6]. Intrinsic magnetic materials, such as metal oxides (e.g., Fe_3_O_4_, γ-Fe_2_O_3_), possess high magnetism and chemical stability. However, their adsorption performance is constrained by an inherently low specific surface area and insufficient active surface sites [7,8]. To overcome the adsorption limitations of intrinsic magnetic materials, researchers have adopted composite strategies to prepare composite magnetic materials. This involves combining the magnetic component with high-surface-area materials or natural polymers, thereby enhancing adsorption capacity, selectivity, and regeneration performance [9]. Nevertheless, this strategy still faces practical challenges, including immature large-scale synthesis processes, unclear selective adsorption mechanisms in complex multi-pollutant systems, and poor structural stability during long-term use. Furthermore, the existing research predominantly focuses on performance optimization at the laboratory scale, lacking systematic life cycle assessments encompassing material sourcing, synthesis energy consumption, and recycling efficiency.

Against this backdrop, this review systematically summarizes recent advances in the preparation methods of various magnetic adsorbents and their application progress in environmental pollutant removal. It critically analyzes performance optimization strategies for composites and discusses key aspects such as interface design, adsorption mechanisms, and large-scale production bottlenecks for magnetic carbon-based materials, natural mineral-based composites, and natural polymer composites. The aim is to provide a theoretical foundation and technical support for the development and application of high-performance magnetic adsorbents (Table 1).

## 2. Synergistic Mechanisms in Magnetic Adsorbents

The core functionality of magnetic adsorbents relies on the multilevel synergy between magnetic components and functional carrier materials. The magnetic component provides strong magnetism (e.g., ferrimagnetism or ferromagnetism), enabling rapid solid-liquid separation post-adsorption. Meanwhile, the functional carrier material offers a large specific surface area, abundant pore structures, and specific functional groups that serve as active adsorption sites. Consequently, the selection of functional carrier materials is pivotal in fabricating magnetic adsorbents.

In carbon-based composites, for instance, the introduction of magnetic particles prevents carbon material agglomeration while exposing more active sites through increased surface area [10]. Simultaneously, the carbon-based carrier protects magnetic components from oxidative corrosion, thereby extending material lifespan. As demonstrated, the π-π conjugation systems in graphene oxide’s aromatic domains and carbon nanotube walls can form hydrogen-bond networks or electron-transfer channels with surface functional groups (e.g., hydroxyl, carboxyl) of magnetic components, significantly enhancing complexation capability toward heavy metal ions and π-π stacking interactions with organic pollutants [11].

Beyond carbon-based materials, inorganic mineral carriers with layered or fibrous structures provide stable platforms for magnetic particle loading. For example, montmorillonite’s high ion-exchange capacity and sepiolite’s Si-OH surface sites provide stable platforms with unique spatial structures (e.g., interlayer galleries or fibrous channels) for accommodating magnetic particles [12,13]. Laponite’s negatively charged surface electrostatically interacts with positively charged Fe_3_O_4_, optimizing particle dispersion [14]. These interactions allow composites to simultaneously leverage mineral–ion exchange and magnetic surface coordination effects during adsorption.

Recent studies further indicate natural polymer materials as suitable carriers for magnetic composites. Their synergistic mechanisms primarily involve dynamic functional coupling: functional groups such as hydroxyls in cellulose, phenolic groups in lignin, and amino groups in gelatin serve dual roles as active sites for pollutant adsorption and anchoring points (via coordination bonds or hydrogen bonds) for magnetic particles, inhibiting their leaching [15]. Conversely, magnetic particle incorporation enhances the mechanical stability of polymer networks through techniques like cyclic freeze–thaw processing [16], while their intrinsic magnetic responsiveness addresses the separation challenges inherent to biomass adsorbents (Table 2).

Building upon this synergistic foundation, Section 3, Section 4 and Section 5 will systematically examine the design principles, synthesis methodologies, and structure–property relationships of magnetic composites based on carbonaceous, mineral, and polymeric carriers, respectively.

## 3. Magnetic Carbon-Based Composites

Magnetic carbon-based composite adsorbents represent a class of functional materials that integrate magnetic nanoparticles with carbon-based materials, such as activated carbon, carbon nanotubes, and graphene. These composites synergistically combine the high specific surface area and rich surface chemistry of carbon materials with the facile separation characteristics of magnetic components. Consequently, they are extensively applied for the efficient removal of heavy metal ions, organic pollutants, and gas molecules from aqueous environments, positioning them as a prominent research focus in the adsorption field.

### 3.1. Activated Carbon

Activated carbon (AC) is a carbonaceous material characterized by a highly developed porous structure. It is typically prepared from carbon-rich biomass feedstocks (e.g., wood, coconut shells, coal) via physical or chemical activation methods [17]. Traditional AC adsorbents, including powdered activated carbon and granular activated carbon, rely primarily on their inherent high specific surface area and microporous structure for pollutant removal [18]. Owing to their excellent adsorption performance and stable physicochemical properties, AC finds widespread application in water treatment. Magnetic activated carbon (MAC) is a functionalized material prepared by compositing magnetic materials with AC [19]. Although AC itself is not typically classified as a nanomaterial, the introduction of nano-magnetic components through composite strategies, or the use of ultrafine AC particles obtained via processes like ball milling or derived from nanocellulose, imparts significant nanoscale characteristics and interfacial effects within MAC. Primary preparation methods for MAC include impregnation-pyrolysis, hydrothermal synthesis, and mechanical ball milling.

Impregnation-Pyrolysis: This common and straightforward method involves impregnating the biomass feedstock in a solution containing magnetic precursors (metal ions), followed by solvent removal and pyrolysis treatment (300–1000 °C) under an inert atmosphere [20]. For instance, Wu et al. [21] prepared MAC by immersing alkaline lignin as biomass raw material in a solution containing iron nitrate and zinc nitrate, and heat treatment under a 600 °C nitrogen atmosphere. The advantage lies in achieving simultaneous magnetization and pyrolysis, ensuring the composite possesses excellent adsorption performance and stable physicochemical properties. However, Cai et al. [22], while preparing MAC from peanut shells via impregnation-pyrolysis, noted that the high-temperature process is energy-intensive and prone to generating toxic gases like CO and combustible gases such as H_2_, conflicting with green chemistry principles. Furthermore, recent studies reveal that excessively high pyrolysis temperatures not only cause the collapse of the AC pore structure but also promote the conversion of iron ions to FeO, weakening magnetism and reducing regeneration capacity [23].

Hydrothermal Synthesis: Compared to impregnation-pyrolysis, hydrothermal synthesis offers milder reaction conditions, eliminating the need for strong alkalis or reducing agents. It involves composite formation in a hydrothermal reactor at lower temperatures (100–300 °C). Lv et al. [24] prepared MAC at 200 °C and found that excessive magnetic component loading could block pore channels, reducing the number of micropores and mesopores. Conversely, appropriately reducing the magnetic content enhanced the MAC’s adsorption capacity for sulfamethoxazole. Zhao et al. [25] modified coconut shell AC hydrothermally and found that optimal adsorption performance for amoxicillin was achieved at a treatment temperature of 160 °C and a raw material particle size of 80–120 mesh, with efficiency significantly influenced by both parameters. Although hydrothermal conditions are mild, the method exhibits high sensitivity to process parameters, requires long reaction times, often yields products with weaker magnetic stability, and traditional heating can lead to uneven magnetic particle size and distribution. Future optimization could leverage microwave-assisted heating technology for precise control over particle size and dispersion [26].

**Figure 1:**
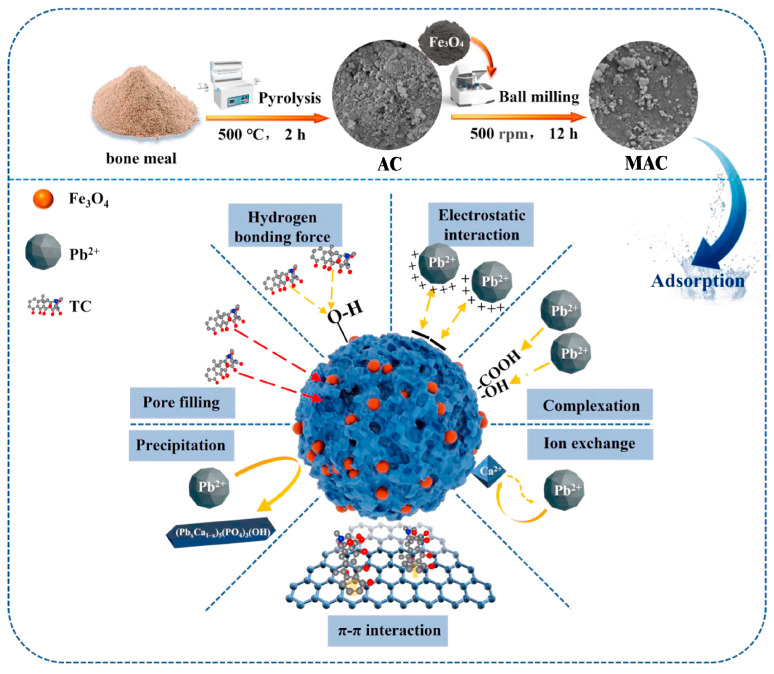
Schematic of ball-mill synthesis of MAC and adsorption mechanism for Pb(II) and TC. Reproduced with permission from ref. [28]. Copyright 2023, Elsevier.

Mechanical Ball Milling: This technique is increasingly applied to MAC preparation. It utilizes the mechanical energy of ball milling equipment to induce solid-state reactions between magnetic substances and AC, enabling the economical and environmentally friendly synthesis of nanoscale ultrafine composites [27]. Qu et al. [28] successfully prepared Fe_3_O_4_-loaded bone-derived biochar via ball milling (Figure 1), demonstrating that the process not only facilitates metal oxide loading but also significantly enhances the material’s adsorption performance for Pb(II) and tetracycline. Asma et al. [29], investigating methylene blue adsorption by AC derived from mangosteen peel, observed improved adsorption capacity with decreasing ball-milled particle size. Li et al. [30] found that ball milling increased AC’s adsorption capacity for methylene blue by an average of three-fold compared to the non-ball-milled AC. Notably, while the incorporation of magnetic Fe_3_O_4_ nanoparticles enabled convenient magnetic separation, it did not significantly enhance the adsorption capacity for MB beyond the improvement already achieved by ball milling the AC alone. The high adsorption capacity is attributed to increased surface area, open pore structure, functional groups, and aromatic C=C bonds, promoting π-π and electrostatic interactions.

The core advantage of MAC lies in “high adsorption + easy recovery.” However, most existing studies focus on adsorbing single pollutants like a specific heavy metal or antibiotic. Real wastewater often contains complex mixtures like organic-heavy metal co-contaminants, where pollutants may compete for adsorption sites. Enhancing selectivity through surface functional group design is crucial. Furthermore, current MAC preparation remains largely confined to small-scale laboratory synthesis. Scaling up faces challenges such as uneven distribution of magnetic particles and significant batch-to-batch variations. Additionally, the long-term leaching of magnetic components and pore structure collapse during recycling requires systematic evaluation (Table 3).

### 3.2. Carbon Nanotubes

Carbon nanotubes (CNTs), with their large specific surface area and hollow internal structure, provide abundant active sites, demonstrating significant potential for adsorbing organic and inorganic pollutants from water [31]. The specific arrangement of carbon atoms in CNTs also confers aromatic properties, facilitating π-π interactions [32]. Magnetic carbon nanotubes (MCNTs), as functional materials combining magnetic responsiveness with nanostructural characteristics [33], are primarily prepared through strategies focused on loading magnetic components. Current research emphasizes chemical modification, in situ synthesis, and physical compounding techniques.

**Figure 2:**
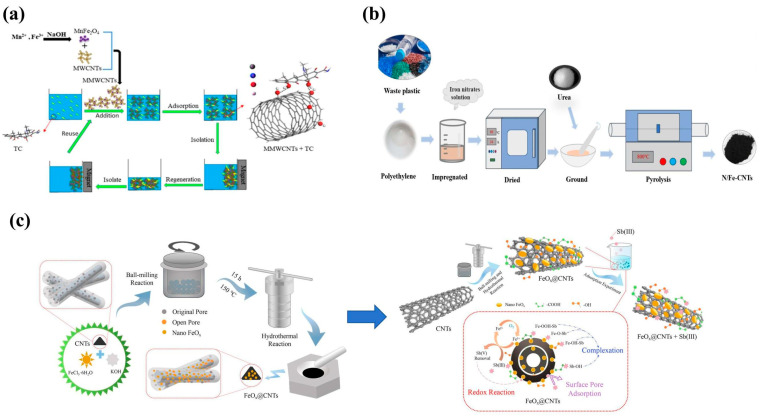
MCNT preparation methods: (**a**) Co-precipitation. Reproduced with permission from ref. [34]. Copyright 2021, Elsevier; (**b**) in situ synthesis. Reproduced with permission from ref. [38]. Copyright 2024, Elsevier; (**c**) ball milling-hydrothermal two-step synthesis. Reproduced with permission from ref. [40]. Copyright 2022, Elsevier.

Chemical Modification: This common strategy anchors magnetic nanoparticles onto the CNT surface via co-precipitation or solvothermal methods. For example, Zhao et al. [34] loaded MnFe_2_O_4_ particles onto CNTs via co-precipitation (Figure 2a). The resulting magnetic multi-walled carbon nanotubes exhibited significant magnetic separability, high adsorption capacity for tetracycline, and retained ~70% removal efficiency after six regeneration cycles. Gao et al. [35] modified multi-walled CNTs with co-precipitated Fe_3_O_4_, finding the material could activate persulfate to degrade bisphenol A and p-chlorophenol over a wide pH range, with degradation efficiencies of 90% and 88%, respectively. While simple, this method can cause magnetic particle agglomeration, and harsh acid/base treatments may damage the CNT wall structure. Future efforts could employ milder oxidants to mitigate wall damage.

In situ Synthesis: This method embeds magnetic components directly during CNT growth by pyrolyzing iron-containing precursors (e.g., ferrocene, ferric nitrate) with a carbon source, allowing control over magnetic particle size distribution by adjusting water/structure-directing agent ratios [36]. Commercially, MCNTs are often prepared by in situ thermal decomposition of magnetic precursors in ethylene glycol onto multi-walled CNTs. Gabriel et al. [37] used this method to obtain magnetic nanomaterials for sampling and purifying trace levels of (fluoro)quinolones in human blood before UPLC analysis. This approach can also be applied to synthesize MCNTs directly from carbon sources. Cheng et al. [38] ground a mixture of urea and dried waste plastic, and an iron nitrate solution, and heated it at 800 °C for 2 h in a resistance furnace under nitrogen (Figure 2b). The residue was N/Fe-CNTs. Adsorption tests confirmed that nitrogen groups and magnetism within the CNTs facilitated Cr(VI) removal via complexation and reduction reactions. In situ synthesis enables precise control over magnetic particle crystallinity and size, but involves high process complexity. For instance, ferrocene pyrolysis requires strict control of carbon/iron ratios to avoid non-magnetic impurities like α-Fe, and the reaction atmosphere significantly affects CNT graphitization [39]. Future work could utilize in situ characterization techniques like combined XRD-TGA to optimize process parameters and minimize nanotube defects.

Physical Compounding: Primarily referring to ball milling, this method combines pre-synthesized magnetic particles with CNTs via mechanical force or interfacial interactions. Cheng et al. [40] synthesized a novel nanoscale iron oxide-modified CNT composite (FeOx@CNTs) using a combined ball-milling–hydrothermal two-step method (Figure 2c). Compared to pristine CNTs, FeOx@CNTs possessed a relatively larger specific surface area, more oxygen-containing functional groups, and more nano-FeO_x_, providing more adsorption sites for Sb(III) removal. Adsorption tests confirmed a 42.9-fold higher Sb(III) adsorption capacity under specific conditions. Dilermando et al. [41] experimentally determined that ball milling time critically influences interfacial bonding strength, but excessive mechanical action can cause CNT fracture. Future improvements could explore short-duration high-energy ball milling combined with annealing for reinforcement.

Current research challenges involve balancing magnetic loading efficiency with CNT structural integrity. Future strategies may employ core–shell designs (e.g., Fe_3_O_4_@C/CNTs) to optimize this balance. Regarding adsorption performance, the hollow channels of CNTs can directionally enrich pollutants. Molecular dynamics simulations could predict the optimal structural match between tube diameter and pollutant molecule size. Addressing issues like high energy consumption and poor process repeatability in scaled-up production is also essential.

### 3.3. Graphene Oxide

**Figure 3:**
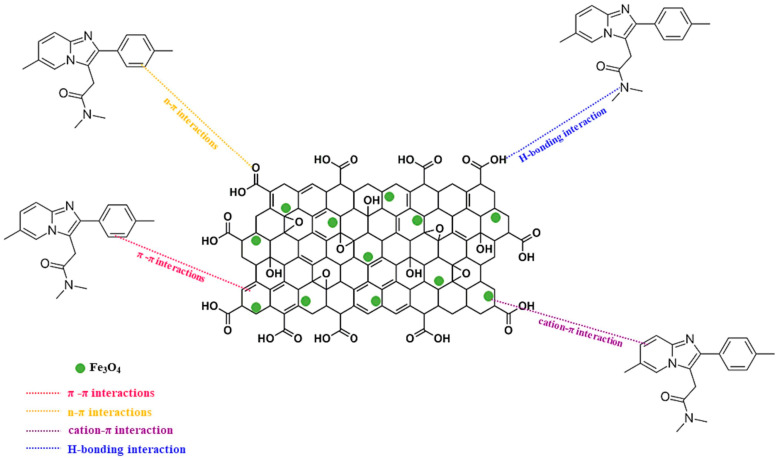
Mechanism of MGO adsorption of aromatic substances. Reproduced with permission from ref. [43]. Copyright 2024, Elsevier.

Graphene oxide (GO), due to its π-π stacking capability, readily adsorbs aromatic substances onto its characteristic conjugated hexagonal ring structure (Figure 3), making it an effective adsorbent for organic pollutants [42]. Magnetic graphene oxide (MGO) combines the high specific surface area and rich functional groups of GO with the rapid separation properties of magnetic nanoparticles. The core of its preparation lies in achieving stable integration between the magnetic component and GO sheets, primarily realized through strategies like chemical co-precipitation, hydrothermal synthesis, and in situ growth.

Chemical Co-precipitation: This is the most prevalent method for MGO preparation. It involves mixing Fe^2+^/Fe^3+^ salt solutions with a GO dispersion, followed by co-precipitation under alkaline conditions to form Fe_3_O_4_/GO composites. Konuk et al. [44] found that increasing the Fe^2+^/Fe^3+^ ratio positively influenced MGO’s size distribution, magnetic properties, morphology, and adsorption–desorption behavior. However, GO sheets, having undergone strong oxidation, may suffer from functional group degradation upon exfoliation, leading to reduced surface area and fewer adsorption sites. To prevent functional group damage, Cheng et al. [45] proposed synthesizing MGO via ultrasound-assisted ammonia co-precipitation, verifying that the resulting material exhibited superior adsorption performance for Cr^3+^. Bulin et al. [46] applied this method to GO hydrogel, and the prepared magnetic GO demonstrated fast adsorption kinetics and high capacity for aqueous Co(II). A limitation is the difficulty in precisely controlling magnetic particle nucleation and growth during co-precipitation, making the process susceptible to particle agglomeration due to pH fluctuations or uneven ion concentration.

Hydrothermal Synthesis: To avoid particle agglomeration, researchers employ hydrothermal synthesis, utilizing high-temperature and high-pressure conditions to promote the oriented growth of magnetic particles on GO sheets. Tran et al. [47] investigated the reaction of Mn^2+^ and Fe^3+^ in a GO solution at 80, 130, and 180 °C, finding that higher hydrothermal temperatures resulted in better crystallinity, magnetism, and As(III) adsorption capacity. Hydrothermal reactions are typically conducted at elevated temperatures. For instance, Gao et al. [48] prepared persimmon tannin-modified MGO (Fe_3_O_4_/PT/GO) nanocomposites at 160 °C for malachite green removal from wastewater, retaining 85.9% of the initial adsorption capacity after five cycles. Adamantia et al. [49] synthesized GO-MnFe_2_O_4_ nanohybrids hydrothermally at 200 °C, with the material maintaining over 65% Congo red removal efficiency after 10 adsorption cycles. While effective against agglomeration, excessively high temperatures may partially disrupt the GO sheet structure [50], and the process itself is energy intensive.

In situ Growth: This method introduces magnetic precursors during GO synthesis to achieve synchronous embedding of the magnetic component. Lu et al. [51] synthesized magnetic nanoparticle-doped GO via both in situ and ex situ methods, finding that the in situ material had a larger specific surface area, more uniform distribution of magnetic nanoparticles on the GO surface, and superior methylene blue dye removal efficiency. Donga et al. [52] synthesized Fe_3_O_4_-GO using in situ precipitation, verifying its better adsorption performance for Rhodamine B and methyl orange. In situ growth achieves atomic-level bonding between magnetic particles and GO but involves complex processes. Furthermore, iron ions may interfere with the oxidation degree of GO, resulting in uneven interlayer spacing.

Self-assembly: This approach composites pre-synthesized magnetic nanoparticles with GO via electrostatic interactions or chemical crosslinking [53]. Chen et al. [54] used a reduction self-assembly strategy to assemble GO and pre-synthesized magnetic components into 3D magnetic fungal hyphae/graphene oxide nanofibers. These were applied to treat high-salinity, extreme-pH wastewater containing various heavy metals from the battery industry, demonstrating good removal efficiency and regeneration performance even under extreme conditions. Zhang et al. [55] chemically crosslinked pre-synthesized modified Fe_3_O_4_ with GO. The resulting material exhibited rapid adsorption kinetics for Co^2+^ ions, reaching equilibrium within 30 s, along with acid tolerance and radiation resistance. This method offers mild conditions and controllable magnetic loading but relies on surface modifiers, potentially introducing impurities or reducing specific surface area.

Despite MGO’s advantages of high adsorption capacity and convenient magnetic recovery in pollutant removal, its practical application faces significant challenges. Unclear selective adsorption mechanisms limit its efficacy in complex multi-pollutant systems like heavy metal-antibiotic coexisting wastewater. The competitive behavior of different pollutants for limited oxygen-containing functional groups and π-π regions needs elucidation through molecular dynamics simulations. Scaling-up bottlenecks are prominent: GO preparation often relies on the high-energy-consumption Hummers method, and fluctuations in Fe^3+^/Fe^2+^ ratios during hydrothermal synthesis leads to batch variations in magnetic saturation strength. Insufficient long-term stability manifests as GO sheet exfoliation during recycling and Fe leaching under acidic conditions. Future research should prioritize several key directions to address these challenges. First, developing material modification strategies is crucial for achieving target-specific adsorption. Second, efforts are needed to design continuous-flow co-precipitation reactors integrated with microwave assistance to address the bottlenecks of high energy consumption and poor batch-to-batch reproducibility associated with conventional methods. Finally, establishing comprehensive life cycle assessment (LCA) models is essential to quantify the environmental benefits and economic costs, encompassing stages from raw material extraction and synthesis energy consumption to end-of-life adsorbent disposal.

## 4. Magnetic Composites Based on Natural Inorganic Mineral Materials

Although magnetic carbon-based composites exhibit excellent adsorption performance and are widely studied for pollutant removal from aqueous solutions, their synthesis and purification can be time-consuming and relatively costly [56]. Consequently, naturally abundant, low-cost, and environmentally friendly natural inorganic mineral materials have garnered increasing attention [57].

Natural inorganic mineral materials primarily include bentonite, montmorillonite, kaolinite, and diatomite. These materials demonstrate significant advantages in preparing magnetic composites due to their unique layered structures, porosity, and abundant surface active sites [58]. By combining natural inorganic minerals with magnetic nanoparticles, functional adsorbents can be fabricated that integrate efficient adsorption performance with facile separation characteristics [59]. This chapter focuses on magnetic composites derived from three inorganic minerals: montmorillonite, sepiolite, and laponite.

### 4.1. Montmorillonite

**Figure 4:**
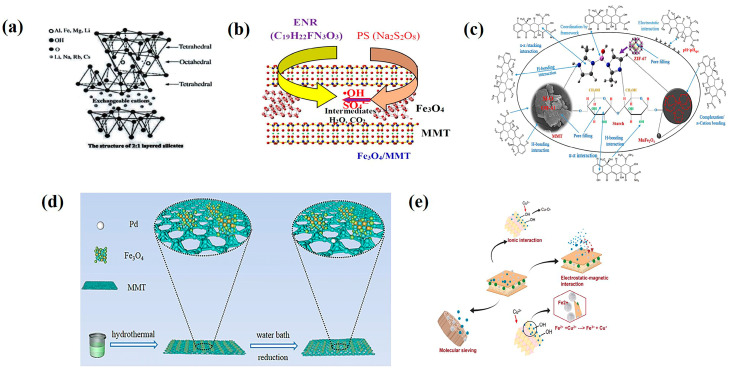
Adsorption mechanisms of magnetic montmorillonite: (**a**) schematic of MMT structure; (**b**) mechanism of tetracycline degradation by the composite. Reproduced with permission from ref. [62]. Copyright 2024, Elsevier; (**c**) mechanism of enrofloxacin degradation by magnetic montmorillonite. Reproduced with permission from ref. [63]. Copyright 2019, Elsevier; (**d**) schematic of Pd@Fe_3_O_4_ magnetic component loading onto montmorillonite. Reproduced with permission from ref. [64]. Copyright 2024, Elsevier; (**e**) mechanism of copper ion adsorption by magnetic montmorillonite. Reproduced with permission from ref. [65]. Copyright 2021, Elsevier.

Montmorillonite (MMT) is a typical layered clay mineral (Figure 4a), characterized by high net negative charge, cation exchange capacity, and layer expansion capability [60]. Magnetic montmorillonite (MMT-M) is a functional material formed by compositing magnetic nanoparticles with MMT. The key to its preparation lies in achieving efficient loading and stable binding of the magnetic component within the interlayers or on the surface of the montmorillonite.

Precipitation Method: This involves introducing Fe^2+^/Fe^3+^ salts into an MMT suspension and generating magnetic montmorillonite via co-precipitation under alkaline conditions. Mekonnen et al. [61] prepared Fe_3_O_4_/MMT composites using both metal reduction and co-precipitation methods. They found that the nanomaterial generated by co-precipitation possessed a larger specific surface area and exhibited superior adsorption performance for methylene blue compared to that from metal reduction. Niroumand [62] used co-precipitation to prepare a starch-containing a magnetic MnFe_2_O_4_-MMT composite. Comparative experiments revealed that the introduction of magnetic particles enhanced the composite’s capacity for removing tetracycline from water (Figure 4b). Peng et al. [63] placed MMT in a ferrous sulfate solution for cation exchange between Fe^2+^ and cations in the MMT interlayers. Under alkaline conditions and continuous air blowing, Fe_3_O_4_/MMT composites formed. They discovered that enrofloxacin adsorbed onto the composite surface was rapidly degraded in situ by reactive radicals generated on the surface in the presence of persulfate (Figure 4c), demonstrating excellent removal efficiency and stability for enrofloxacin. While operationally simple, the precipitation method requires strict control of pH and ionic strength, increasing process complexity. Furthermore, air blowing and alkaline environments can readily oxidize Fe^2+^, compromising magnetic stability.

Hydrothermal Synthesis: This method promotes the in situ growth of magnetic particles on the MMT surface under high-temperature and high-pressure conditions, often used for composite formation or one-step modification. Hou [64] synthesized Pd@Fe_3_O_4_/MMT nanocomposites via a simple hydrothermal method (Figure 4d), finding the material capable of photocatalytic tetracycline degradation. Fatimah [65] observed that Fe_3_O_4_ particles synthesized hydrothermally were smaller than those produced by co-precipitation. A series of adsorption performance tests confirmed that hydrothermal synthesis enhanced the copper ion adsorption capacity of the magnetic montmorillonite nanocomposite (Figure 4e). Chairul et al. [66] successfully loaded amine-functionalized magnetic nanoparticles onto the surface layers of montmorillonite hydrothermally. The composite exhibited stronger adsorption capacity for Pb(II) and As(V) in mixed solutions compared to pristine montmorillonite. However, the high-pressure conditions of hydrothermal reactions may damage the layered structure of montmorillonite, weakening its inherent adsorption advantages. Future research could explore composite processes under milder conditions, such as microfluidic-controlled co-precipitation or low-temperature hydrothermal synthesis, to balance structural integrity and functionalization efficiency.

Existing preparation methods often rely on organic chemical reagents, posing potential secondary pollution risks. Future development could focus on bio-templating methods like microbial mineralization, although the potential secondary risks from microbial metabolites must also be thoroughly assessed. Then, waste-derived MMT carriers, such as industrial bentonite tailings, can be utilized as a breakthrough combining environmental friendliness and economic viability. The environmental cost of bentonite mining should also be quantified in future life cycle studies. Moreover, most adsorption performance research only validates short-term efficacy, neglecting the impact of complex components in real water bodies on material stability. Future work should conduct aging experiments like cyclic adsorption–regeneration and long-term exposure tests, establishing quantitative evaluation standards for magnetic recovery rate and particle detachment rate.

### 4.2. Sepiolite

**Figure 5:**
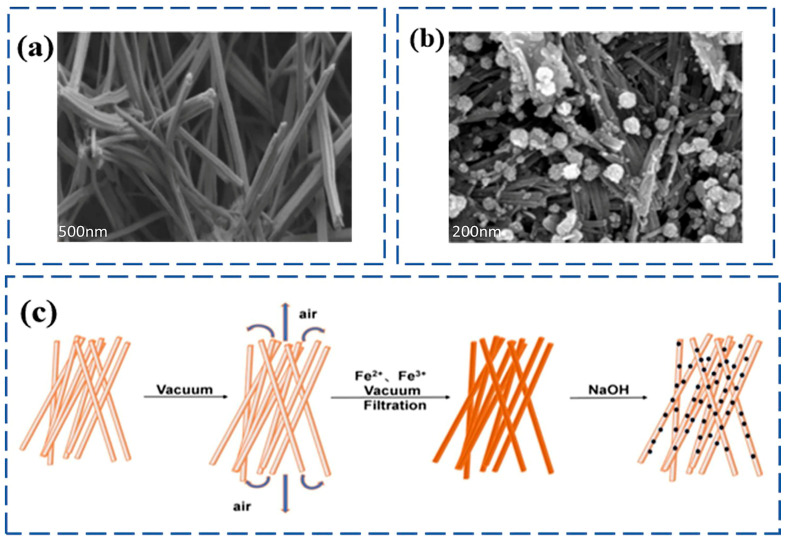
(**a**) SEM image of sepiolite. Reproduced with permission from ref. [71]. Copyright 2025, Elsevier; (**b**) SEM image of MS. Reproduced with permission from ref. [69]. Copyright 2022, Elsevier; (**c**) schematic diagram of vacuum filtration-assisted co-precipitation. Reproduced with permission from ref. [70]. Copyright 2024, Elsevier.

Sepiolite is a natural, economical, and abundant hydrated magnesium silicate clay mineral. Its surface is rich in highly reactive silanol groups (Si-OH), facilitating the selective adsorption of various organic molecules, inorganic ions, and even nanoparticles [67]. Magnetic sepiolite (MS) is a functional material prepared by compositing magnetic nanoparticles with natural sepiolite. Its preparation requires introducing the magnetic component while preserving sepiolite’s fibrous porous structure (Figure 5a). The key challenge lies in avoiding pore blockage or destruction of the sepiolite crystal structure by magnetic particles. Primary preparation methods include co-precipitation, hydrothermal synthesis, and sol-gel processes.

Co-precipitation Method: This is a common approach for MS preparation. Shi [68] used co-precipitation to prepare magnetic sepiolite as a magnetic medium carrier. They found that magnetic sepiolite could wrap, embed, or adsorb onto microplastic surfaces, enabling their magnetic separation from aqueous solutions. Zhu [69] synthesized magnetic Prussian blue nano-adsorbents loaded onto sepiolite (PB/Fe_3_O_4_/SEP) (Figure 5b), where the composite’s high adsorption capacity facilitated the removal of trace Tl(I) from wastewater. To achieve highly monodisperse and stable composites in complex environments, Kang et al. [70] pioneered a vacuum-filtration-assisted co-precipitation strategy (Figure 5c) to synthesize a highly dispersed Fe_3_O_4_ nanoparticle-modified sepiolite composite. Compared to pure Fe_3_O_4_ nanoparticles, pristine sepiolite, and composites prepared by traditional co-precipitation, the resulting material exhibited the highest adsorption capacity for ciprofloxacin. Vacuum-filtration-assisted co-precipitation effectively enhanced the dispersion of Fe_3_O_4_, demonstrating that externally applied physical fields can overcome limitations of traditional co-precipitation. Future research could explore novel co-precipitation strategies like ultrasound assistance or microfluidic technology to further improve the loading uniformity of magnetic particles.

Hydrothermal Synthesis: This method can reduce damage to the sepiolite structure and is often used for compositing magnetic sepiolite with other materials. For example, Su et al. [72] hydrothermally prepared novel core–shell structured Fe_3_O_4_@SiO_2_@BiFeO_3_-sepiolite microspheres. The core–shell structure of Fe_3_O_4_, large specific surface area, and mesoporous channels endowed the photocatalyst with high light utilization, excellent light capture ability, and abundant active reaction sites. The composite exhibited significant photocatalytic degradation performance for ciprofloxacin, tetracycline, and methylene blue, and achieved noticeable decolorization of mixed solutions containing methyl orange, Rhodamine B, and methylene blue under visible light irradiation. Deng [73] synthesized carbon-magnetic modified sepiolite nanocomposites hydrothermally using γ-Fe_2_O_3_, activated sludge biochar, and alkali-modified sepiolite. The material demonstrated excellent adsorption performance for heavy metals like Sb(V), Pb(II), Cd(II), and Zn(II), even when coexisting in contaminated water sources. However, hydrothermal reactions require long durations and precise control of precursor concentration to prevent excessive particle growth.

Sol-Gel Method: This process involves combining a magnetic precursor sol with a sepiolite suspension, followed by gelation, drying, and calcination to obtain MS materials. Xu et al. [74] prepared a novel magnetic sepiolite adsorbent loaded with copper ferrite using the sol-gel method, concluding that the main adsorption mechanisms for Pb(II) removal were electrostatic attraction, ion exchange, and surface complexation. Li et al. [75] successfully fabricated a novel photocatalyst, nano-titanium dioxide (TiO_2_/MMS) loaded magnetic modified sepiolite, with high photoactivity and magnetic separability via sol-gel. They found that the composite significantly enhanced the redox interaction in the simultaneous photocatalytic degradation of the Cr(VI) and 2,4-dichlorophenol (DCP) binary system, achieving removal efficiencies of approximately 100% and 96.9%, respectively. The sol-gel method enables uniform dispersion of magnetic particles and employs relatively low calcination temperatures (<500 °C), effectively protecting sepiolite’s hydroxyl active sites. However, its complex process flow and high cost limit large-scale applications. Future work could explore green sol-gel systems utilizing bio-templating or low-temperature gelation to reduce energy consumption and preserve sepiolite’s active sites.

Current research predominantly focuses on the macroscopic performance of MS, such as adsorption capacity and photocatalytic efficiency, but lacks in-depth analysis of key scientific issues like the loading position of magnetic particles and surface modification strategies. Subsequent studies should combine in situ characterization techniques and theoretical calculations to elucidate the structure–property relationships of MS, providing a basis for rational material design.

### 4.3. Laponite

Laponite is a layered silicate clay whose nanoscale sheet structure and tunable surface charge provide an ideal carrier for loading magnetic components [76]. Magnetic laponite (ML) is a functional nanomaterial prepared by compositing magnetic nanoparticles with laponite, often introduced into hydrogel systems to enhance gel stability. The key to preparing ML lies in achieving uniform dispersion of magnetic particles between the laponite sheets while preventing nanosheet stacking or magnetic agglomeration. The mainstream method is currently co-precipitation.

**Figure 6:**
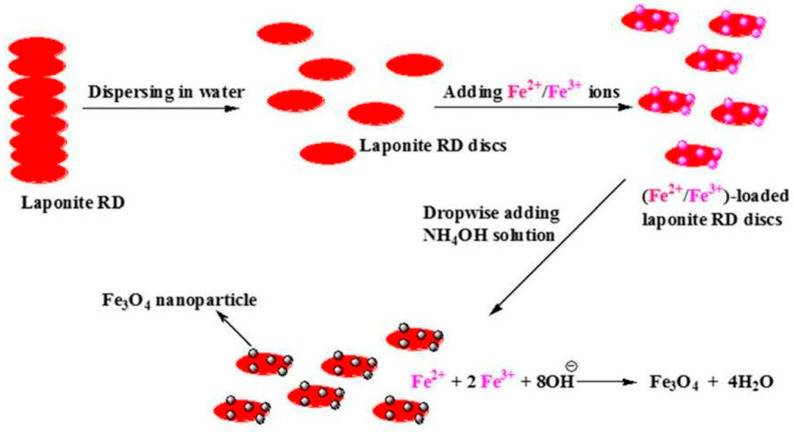
Schematic of ML preparation by co-precipitation. Reproduced with permission from ref. [79]. Copyright 2018, Springer.

Co-precipitation Method: This common strategy involves dispersing laponite in an iron salt solution under an inert atmosphere and adding an alkaline precipitant to generate magnetic particles. Gholam [77,78,79] fixed magnetite nanoparticles onto negatively charged laponite sheets via co-precipitation (Figure 6). Subsequent cyclic freeze–thaw processing yielded magnetic polyvinyl alcohol/laponite composite hydrogels with good reusability and favorable adsorption performance for both methylene blue and cadmium ions. Shiva et al. [80] prepared magnetic chitosan/cellulose/laponite nanocomposite hydrogels via in situ precipitation. Comparative adsorption tests for various ions revealed that the composite hydrogel exhibited high selectivity for Hg(II) ions. While enabling directional loading of magnetic particles, co-precipitation requires long reaction times and strict control of precursor concentration to prevent excessive particle growth. Furthermore, the alkaline environment during co-precipitation may cause partial dissolution of laponite sheet edges, and magnetic particles tend to aggregate on the sheet surface, reducing the number of active sites.

Current research on magnetic laponite is relatively scarce. There is a lack of systematic investigation into how laponite’s nanolayered structure affects the catalytic activity of magnetic particles, or how the distribution of magnetic particles between and on the laponite sheets influences the material’s ion exchange capacity and magnetic separation efficiency. The development of ML is hindered by three main shortcomings: (1) large-scale preparation relies on expensive high-purity laponite, while impurities in natural mineral sources cause fluctuations in composite magnetic responsiveness; (2) poor adaptability to complex water bodies, as high salinity can shield interlayer electrostatic interactions, leading to reduced adsorption capacity; (3) regeneration techniques are rudimentary, with acid/base desorption potentially dissolving the siloxane framework at pH < 2 or pH > 12. Future innovation paths include: ① developing low-cost, high-purity laponite synthesis methods; ② combining electrochemical regeneration with MOF compositing to enhance salt resistance; ③ establishing an ML mineral resource map, assessing regionalized production feasibility using Geographic Information Systems (GIS), and conducting accelerated aging tests over extensive cycles (e.g., >50 cycles) to predict practical service life (Table 4).

## 5. Magnetic Composites Based on Natural Polymer Materials

While natural mineral clays are abundant and low-cost, they often face challenges in regeneration. In contrast, natural polymer materials, derived from biomass resources, offer renewability, biodegradability, and rich surface functional groups (e.g., hydroxyl, amino, carboxyl) [81]. These properties make them environmentally friendly matrix choices for developing magnetic adsorbents [82]. This section focuses on the preparation strategies and application advances of magnetic composites based on cellulose, lignin, and gelatin.

### 5.1. Cellulose

**Figure 7:**
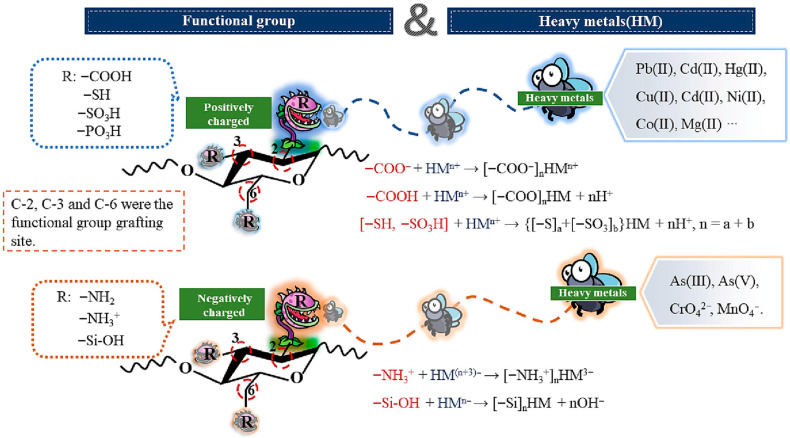
General mechanism of modified nanocellulose for heavy metal removal. Reproduced with permission from ref. [84]. Copyright 2023, Elsevier.

Cellulose is the most abundant natural polymer on Earth. The hydroxyl groups within its linear chain structure can be chemically modified to introduce functional groups, providing active sites for pollutant adsorption [83]. Nanocellulose, in particular, possesses surface hydroxyl groups that are readily complex with metal ions, promoting uniform dispersion of these ions within the cellulose matrix and enhancing adsorption efficiency (Figure 7). Primary preparation methods for magnetic cellulose composites include hydrothermal co-precipitation and self-assembly.

Hydrothermal Co-precipitation: This method involves introducing iron salt solutions into cellulose dispersion and generating magnetic particles via co-precipitation under alkaline or high-temperature/high-pressure conditions. It is often used to utilize cellulose as a linker for multi-material composites. For instance, Zhou et al. [85] first prepared CNF/Fe_3_O_4_ via a one-step hydrothermal method and then compounded it with magnesium hydroxide, using cellulose as the component connecting the magnetic phase and Mg(OH)_2_ to synergistically adsorb cadmium from water. Hydrothermal co-precipitation can also directly optimize cellulose-based adsorbents. Ahmed [86] developed magnetic cellulose nanocrystals from microcrystalline cellulose using a one-step hydrothermal method; after modification, the nanocomposite exhibited significantly enhanced adsorption capacity for doxycycline hydrochloride. Shruthi et al. [87] incorporated magnetic nanoparticles into a hydrogel network of banana pseudostem cellulose fibers grafted with N-hydroxyethyl ethylacrylamide via co-precipitation. Experiments confirmed that the introduction of magnetic particles increased the maximum adsorption capacity for methylene blue and crystal violet from 235 mg/g and 219 mg/g to 320 mg/g and 303 mg/g, respectively. While operationally straightforward, the high crystallinity of cellulose, as the dense, ordered structure of crystalline cellulose, may limit the accessibility of hydroxyl groups and the diffusion of magnetic precursors, hindering the uniform loading of magnetic particles.

Self-Assembly Method: This approach composites magnetic nanoparticles with cellulose via electrostatic interactions or chemical crosslinking.

**Figure 8:**
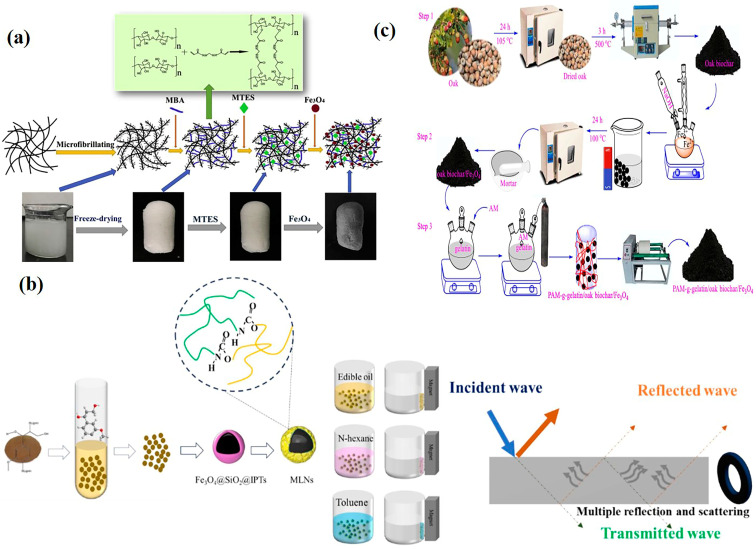
Preparation of magnetic natural polymers: (**a**) process flowchart for preparing magnetic hydrophobic cellulose aerogel. Reproduced with permission from ref. [89]. Copyright 2021, Elsevier; (**b**) hierarchical assembly bonding technology for preparing core–shell magnetic lignin. Reproduced with permission from ref. [95]. Copyright 2025, Elsevier; (**c**) preparation process of gelatin/biochar/Fe_3_O_4_ composite coagulant. Reproduced with permission from ref. [101]. Copyright 2025, Elsevier.

1. Chemical Crosslinking: Utilizes crosslinking agents to anchor magnetic particles onto the cellulose surface. Liu et al. [88] used glutaraldehyde as a crosslinker to prepare polyethyleneimine-modified magnetic sugarcane bagasse cellulose film. This film material achieved 92.63% removal efficiency for ibuprofen, demonstrating high hydrophilicity, thermal stability, non-toxicity, and maintained ~96% removal efficiency after 17 cycles. He et al. [89] employed N, N′-methylenebisacrylamide (MBA) as a crosslinker to prepare magnetic and hydrophobic cellulose Fe_3_O_4_ aerogels (Figure 8a). The aerogel retained its original fluffy and porous structure, exhibiting excellent superhydrophobicity and superparamagnetism. After 10 adsorption–recovery cycles for oil, the aerogel maintained a high oil adsorption rate. However, the use of crosslinking agents might block cellulose pores, reducing specific surface area.
2. Physical Coating: Combines pre-synthesized magnetic particles with cellulose via ultrasonic dispersion or electrostatic adsorption. For example, Barzegarzadeh et al. [90] compared magnetic cellulose/Al-MOF prepared with and without ultrasound, finding they followed the Freundlich and Langmuir models, respectively. Ultrasound increased the adsorption capacity for doxorubicin from 96 mg/g to 108 mg/g through cavitation effects. This method avoids complex chemical modification but suffers from weaker physical binding strength, potentially leading to particle detachment.

Magnetic cellulose shows significant potential in heavy metal and organic pollutant remediation. However, its selectivity in coexisting in heavy metal–dye systems requires further exploration. Traditional preparation processes often cause uneven dispersion of nanocellulose, leading to magnetic particle agglomeration, while emerging physical coating techniques suffer from low composite binding strength, resulting in magnetic component detachment. Future research could explore bio-templating methods (e.g., using bacterial cellulose) to construct ordered pore structures and enhance adsorption kinetics.

### 5.2. Lignin

Lignin is an aromatic natural polymer, rich in functional groups such as phenolic hydroxyls and methoxy groups, conferring strong affinity for heavy metal ions and organic pollutants [91]. The primary preparation strategy for magnetic lignin composites is self-assembly.

Self-Assembly Method: This technique exploits electrostatic interactions or hydrogen bonding between lignin and magnetic particles for self-assembly. Tan et al. [92] prepared a lignin-based magnetic biosorbent by placing the lignin fraction from hydrogen peroxide-treated corn stover (a waste stream from bioethanol production via delignification) and Fe_3_O_4_ inorganic magnetic particles together in an alkaline solution under stirring. The material demonstrated efficient removal performance for both Pb^2+^ and Cu^2+^. Wang et al. [93] synthesized a magnetic chitosan-lignin composite AL-CTS@MNPs via a one-pot method by reacting all components (magnetic particles, chitosan, lignin) in a three-neck flask. The material exhibited high selective adsorption for the anionic dye methyl orange but no adsorption for the cationic dye methylene blue. It showed relatively good cycling stability, and its micron-sized particles facilitated convenient separation as a magnetic chromatographic column filler. Zhao et al. [94] prepared superparamagnetic composites via the self-assembly of enzymatic lignin (or its modified derivatives) and γ-Fe_2_O_3_ nanoparticles under electrostatic interactions. The synthesized magnetic acetylated modified lignin possessed a well-defined spherical structure, exhibited rapid adsorption capacity for both cationic and anionic dyes, and maintained >90% removal efficiency across a wide pH range and in high-concentration (0.5 mol/L) inorganic salt solutions. While the synthesis conditions are mild, the uneven molecular weight distribution of lignin may affect assembly efficiency. To address this, Bai et al. [95] employed a novel hierarchical assembly bonding technique (Figure 8b), successfully assembling more lignin onto the Fe_3_O_4_ particle surface and combining magnetic lignin nanoparticles with a core–shell structure. These particles exhibited excellent adsorption performance in toluene, cooking oil, and n-hexane environments. Additionally, the particles possessed electromagnetic wave absorption capability, synergistically accelerating the oil absorption process.

Lignin, with its rich aromatic structure and active functional groups like phenolic hydroxyls, provides a highly promising green matrix for constructing high-performance magnetic adsorbents. However, current research often involves chemically impregnating and pyrolyzing lignin biomass into biochar before composite adsorption [96], with relatively fewer studies utilizing lignin alone as the adsorbent. Consequently, there is insufficient depth in the mechanistic analysis of adsorption between lignin’s specific functional groups and pollutants, as well as the synergistic efficiency between lignin and magnetic particles. Future research should focus on: (i) improving the homogeneity and reactivity of forces through structural modification; (ii) enhancing the molecular level adsorption mechanisms through advanced characterization and simulations; and (iii) developing mild synthesis routes to create efficient and mechanically robust adsorbent materials to advance practical applications.

### 5.3. Gelatin

Gelatin is a biological macromolecule obtained from the hydrolysis of collagen. The amino and carboxyl groups within its molecular chains can capture pollutants via coordination or electrostatic interactions [97]. The preparation of magnetic gelatin composites primarily relies on physical crosslinking.

Physical Crosslinking: This method immobilizes magnetic particles through ionic crosslinking or cyclic freeze–thaw processes. Huang et al. [98] prepared polyvinyl alcohol/alginate/gelatin/quaternary ammonium chitosan/Fe_3_O_4_ particle hybrid hydrogel beads using a combined Ca^2+^ and tannic acid freeze–thaw method. They systematically studied and compared the impact of the magnetic component’s presence on the adsorption capacity for methyl orange, tetracycline, and Cr(VI). Results showed similar adsorption capacities for methyl orange and Cr(VI) between materials with and without Fe_3_O_4_, but the presence of Fe_3_O_4_ significantly enhanced tetracycline adsorption. Cui et al. [99] prepared magnetic gelatin/carboxymethyl cellulose cryogels via dual crosslinking by incorporating transglutaminase as a crosslinker, followed by immersion in calcium chloride. They found that the amount of introduced nano-magnetic particles influenced the hydrogel’s porosity, thermal stability, and adsorption performance for Congo red. Anulekshmi et al. [100] used glutaraldehyde as a crosslinker to enhance biocompatible gelatin hydrogel with magnetite nanoparticles. They discovered that the large specific surface area of the magnetic particles combined with gelatin led to the formation of mesoporous channels within the hydrogel, enhancing its adsorption performance for Cr(VI). Yao et al. [101] proposed a novel composite coagulant (Figure 8c) composed of polyacrylamide-grafted gelatin, oak-derived biochar, and Fe_3_O_4_ magnetic nanoparticles for the effective removal of turbidity and microcystin-LR from contaminated water. Grafting functional monomers can enhance the interfacial bonding between gelatin and magnetic particles. Although crosslinking methods are simple, the swelling characteristics of gelatin during preparation may cause structural collapse.

The application of gelatin-based magnetic composites faces significant limitations: lack of selectivity hampers control over competitive adsorption in coexisting pollutant systems, and the contribution mechanism of functional groups remains unclear; scalability obstacles arise from the thermosensitivity of gelatin raw materials and difficulties in controlling crosslinking degree during continuous production; stability concerns manifest as low mechanical strength of wet-state gels, relatively high magnetic particle detachment rates after multiple cycles, and a conflict between biodegradability and long-term usage requirements. To address the aforementioned challenges of selectivity, scalability, and stability, breakthrough directions should focus on: ① introducing molecularly imprinted layers to enhance selectivity; ② developing low-temperature photopolymerization processes for one-step molding of gelatin-magnetic particles; and ③ designing “repairable networks” and magnetic core–shell structures to reduce particle detachment. There is an urgent need for research tracing the performance differences between mammalian and fish-source gelatin using metabolomics to assess the ecological toxicity of adsorbent degradation products, thereby refining their greenness evaluation (Table 5).

## 6. Conclusions

MNAs, leveraging their magnetically driven separation characteristics and designable functional interfaces, have emerged as a prominent research focus in the remediation of aqueous pollutants. This review systematically examines the composite strategies involving three categories of carrier materials—carbon-based, inorganic minerals, and natural polymers—with the following key conclusions:

- The integration of magnetic components with carriers induces synergistic enhancement in the composites. The carriers provide a large specific surface area and active adsorption sites, while the magnetic components confer magnetic responsiveness. The chemical bonding, electrostatic self-assembly, and spatial confinement between them significantly improve the structural stability of the composites and their efficiency in capturing pollutants.
- The preparation process substantially influences the performance of magnetic composites. Techniques such as hydrothermal synthesis, co-precipitation, in situ synthesis, and auxiliary methods like microwave/ball milling each possess distinct advantages and disadvantages. Furthermore, different types of carrier materials are suited to specific magnetic loading strategies.
- However, the field still faces significant challenges:
- Unclear selective adsorption mechanisms hinder effective performance in multi-pollutant systems due to competitive adsorption.
- Prominent bottlenecks in scalable production manifest as high energy consumption and poor batch-to-batch reproducibility.
- Absence of comprehensive LCA neglects the environmental costs associated with raw material extraction, synthesis processes, and disposal of spent adsorbents.

## 7. Future Perspectives

Future research should prioritize the following directions:

- Enhance targeted adsorption capabilities in complex systems through molecular imprinting technology and biomimetic surface modification.
- Develop green mining practices for mineral resources and synthesis routes utilizing biomass-derived carriers to achieve sustainable large-scale production.

Establish integrated ‘adsorption-regeneration-resource recovery’ systems utilizing electrochemical desorption technology. Coupled with rigorous lifecycle assessment models, these systems should quantify the environmental benefits and economic costs of MNA implementation.

Addressing these challenges and pursuing these future directions through interdisciplinary innovation and deep industry-=–academia–research integration will be crucial for translating magnetic adsorbents from laboratory prototypes into practical engineering solutions, offering efficient and sustainable strategies for industrial wastewater treatment.

## Figures and Tables

**Table 1:** Comparison of different carrier materials for magnetic nanocomposite adsorbents.

Carrier Category	Key Advantages	Main Limitations	Typical Target Pollutants
Carbon-based materials	high specific surface area rich porosity strong modifiability	relatively high cost synthesis may involve toxic chemicals	dyes antibiotics heavy metals
Inorganic mineral materials	low cost abundant reserves high ion exchange capacity	poor regenerability performance susceptible to environmental pH	heavy metal ions cationic dyes
Natural polymer materials	renewable biodegradable abundant functional groups	low mechanical strength poor stability in complex water matrices	various heavy metals and organic pollutants

**Table 2:** Comparison of synergistic mechanisms in magnetic nanocomposite adsorbents with different carrier matrices.

Carrier Category	Typical Carriers	Core Synergistic Mechanisms	Key Influencing Factors	Performance Enhancement Effect
Carbon-based materials	Activated carbon Carbon nanotubes Graphene oxide	π-π stacking/electrostatic attraction Magnetic particle-oxygen functional group H-bond networks Fe^2+^/Fe^3+^ reduction effects	Graphitization degree, functional group density, magnetic particle loading position	Increased adsorption capacity
Inorganic mineral materials	Montmorillonite Sepiolite Laponite	Cation exchange fixation of Fe^2+^/Fe^3+^ Binding with surface silanol groups Interlayer confinement inhibiting particle growth	Layer charge density, cation exchange capacity, mineral pretreatment	Selective adsorption of heavy metal ions
Natural polymer materials	Cellulose Lignin Gelatin	Functional group coordination bonding π-π interactions via lignin aromatic structures Polymer network encapsulation prevents detachment	Functional group density, crosslinking degree, molecular weight, magnetic particle surface modification	Enhanced anti-salinity interference capability

**Table 3:** Comparison of synthesis methods for MAB.

Synthesis Method	Key Process Conditions	Advantages	Disadvantages	References
Impregnation-Pyrolysis	300–1000 °C, inert atmosphere	Simultaneous carbonization and magnetization, stable material properties	High energy consumption, may generate harmful gases, pore collapse	[20,21,22,23]
Hydrothermal Synthesis	100–300 °C, autoclave	Mild reaction conditions, controllable morphology	Long reaction time, sensitive to parameters, sometimes poor magnetic stability	[24,25,26]
Mechanical Ball Milling	Room temperature, mechanical force	Simple operation, green and economical, potential for scalability	May cause structural damage, potentially weak interfacial bonding	[27,28,29,30]

**Table 4:** Adsorption performance and practical challenges of magnetic mineral composites.

Mineral Carrier	Typical Composite	Adsorption Capacity (Example)	Adsorption Mechanism	Practical Application Challenges
Montmorillonite	Fe_3_O_4_/MMT	TC: ~285 mg/g [58]	ion exchange surface complexation catalytic degradation	Layered structure easily damaged under high pressure, Fe^2+^ prone to oxidation
Sepiolite	Fe_3_O_4_/SEP	CIP: ~100 mg/g [66]	complexation with surface groups channel capture	Fibrous structure prone to pore blockage, performance varies with mineral purity
Laponite	PVA/Laponite/Fe_3_O_4_	Cd^2+^: ~90 mg/g [75]	interlayer confinement electrostatic attraction	High cost, weak resistance to ionic interference, difficult regeneration

**Table 5:** Functionalization strategies and effects for magnetic natural polymer adsorbents.

Polymer Matrix	Functionalization Strategy	Effect	New Functionality Introduced	References
Cellulose	Grafting polyethyleneimine (PEI)	Significantly enhanced adsorption capacity for ibuprofen	Introduces amine groups for higher affinity to specific pollutants	[88]
Lignin	Acetylation modification	Rapid adsorption capacity for both cationic and anionic dyes	Modifies hydrophilicity/hydrophobicity, enhances structural stability	[94]
Gelatin	Blending with alginate/PVA	Forms robust hydrogel beads for easy separation	Improves mechanical properties, enables multi-network synergistic adsorption	[98]
